# Perioperative antibiotic prophylaxis in the treatment of acute cholecystitis (PEANUTS II trial): study protocol for a randomized controlled trial

**DOI:** 10.1186/s13063-017-2142-x

**Published:** 2017-08-23

**Authors:** Charlotte S. Loozen, Hjalmar C. van Santvoort, Antoinette A. W. van Geloven, Grard A. P. Nieuwenhuijzen, Philip R. de Reuver, Mark H. G. Besselink, Bart Vlaminckx, Johannes C. Kelder, Catherijne A. J. Knibbe, Djamila Boerma

**Affiliations:** 1 Department of Surgery, St. Antonius Hospital, Koekoekslaan 1, Postbus 2500, 3430 EM Nieuwegein, The Netherlands; 2 Department of Surgery, Tergooi Hospital, Hilversum, The Netherlands; 3 Department of Surgery, Catharina Hospital, Eindhoven, The Netherlands; 4 Department of Surgery, Radboud University Medical Center, Nijmegen, The Netherlands; 5 Department of Surgery, Academic Medical Center, Amsterdam, The Netherlands; 6 Department of Medical Microbiology, St. Antonius Ziekenhuis, Nieuwegein, The Netherlands; 7 Department of Clinical Epidemiology, St. Antonius Ziekenhuis, Nieuwegein, The Netherlands; 8 Department of Clinical Pharmacology, St. Antonius Ziekenhuis, Nieuwegein, The Netherlands

## Abstract

**Background:**

The additional value of perioperative antibiotic prophylaxis in preventing infectious complications after emergency cholecystectomy for acute cholecystitis is a much-debated subject in the surgical community. Evidence-based guidelines are lacking, and consequently the use of antibiotic prophylaxis varies greatly among surgeons and hospitals. Recently, high-level evidence became available demonstrating that *postoperative* antibiotic prophylaxis in patients with acute cholecystitis does not reduce the risk of infectious complications. *Preoperative* antibiotic prophylaxis in relation to the risk of infectious complications, however, has never been studied.

**Methods:**

The PEANUTS II trial is a randomized, controlled, multicenter, open-label noninferiority trial whose aim is to determine the utility of *preoperative* antibiotic prophylaxis in patients undergoing emergency cholecystectomy for acute calculous cholecystitis. Patients with mild or moderate acute cholecystitis, as defined according the Tokyo Guidelines, will be randomly assigned to a single preoperative dose of antibiotic prophylaxis (2000 mg of first-generation cephalosporin delivered intravenously) or no antibiotic prophylaxis before emergency cholecystectomy. The primary endpoint is a composite endpoint consisting of all postoperative infectious complications occurring during the first 30 days after surgery. Secondary endpoints include all the individual components of the primary endpoint, all other complications, duration of hospital stay, and total costs. The hypothesis is that the absence of antibiotic prophylaxis is noninferior to the presence of antibiotic prophylaxis. A noninferiority margin of 10% is assumed. With a 1-sided risk of 2.5% and a power of 80%, a total of 454 subjects will have to be included. Analysis will be performed according to the intention-to-treat principle.

**Discussion:**

The PEANUTS II trial will provide evidence-based advice concerning the utility of antibiotic prophylaxis in patients undergoing emergency cholecystectomy for acute calculous cholecystitis.

**Trial registration:**

Netherlands Trial Register, NTR5802. Registered on 4 June 2016.

**Electronic supplementary material:**

The online version of this article (doi:10.1186/s13063-017-2142-x) contains supplementary material, which is available to authorized users.

## Background

Acute calculous cholecystitis is a frequently encountered disease in surgical practice that generally mandates emergency cholecystectomy. Although this is considered to be a low-risk procedure, the complication rate is not negligible. The most common complication is a postoperative infection, either at the surgical site or a distant site, occurring in approximately 17% of patients [1]. As such, many patients receive antibiotic prophylaxis before cholecystectomy, often to be continued for several postoperative days, in order to reduce postoperative infectious complications.

The Working Party on Antibiotic Policy in the Netherlands (Stichting Werkgroep Antibioticabeleid) issued guidelines for perioperative antibiotic prophylaxis in Dutch hospitals [2]. According to these guidelines, perioperative antibiotic prophylaxis is recommended for surgical procedures with a moderate or high risk of postoperative infections, including biliary surgery. Prophylaxis given within 2 h before incision appears to be most effective [2]. Recommended is single-dose prophylaxis, not only because it has proven to be as effective as multiple-dose prophylaxis but also for reasons of cost-effectiveness and prevention of bacterial resistance. The Surgical Infection Society and the Infectious Diseases Society of America, as well as the Tokyo Guidelines, also recommend antimicrobial prophylaxis for patients undergoing cholecystectomy for acute cholecystitis [3, 4]. These recommendations, however, are based on low-quality evidence, and therefore the actual effect of perioperative antibiotic prophylaxis remains unclear. Consequently, the use of antibiotic prophylaxis in the treatment of acute cholecystitis is highly variable among surgeons and hospitals.

In patients undergoing *elective* cholecystectomy for uncomplicated cholelithiasis, high-level evidence is available demonstrating that prophylactic antibiotics do not reduce the incidence of postoperative infections [5–9]. For this indication, the use of perioperative antibiotic prophylaxis is discouraged. According to a recent randomized controlled trial, also in *emergency* cholecystectomy the continuation of antibiotic prophylaxis after surgery is disputable [1]. This study demonstrated that postoperative antibiotic prophylaxis (in addition to a single prophylactic dose prior to surgery) in patients with mild and moderate acute cholecystitis did not reduce the risk of infectious complications. Antibiotic prophylaxis after cholecystectomy for acute cholecystitis may therefore be omitted. The remaining question is whether a single preoperative dose of antibiotic prophylaxis is beneficial in patients undergoing emergency cholecystectomy for acute cholecystitis. This has never been studied.

If the present study demonstrates that omitting antibiotic prophylaxis does not increase the postoperative infection rate, the use of antibiotics for this indication can be dropped as a whole. If so, the role of antibiotic prophylaxis in surgery of the entire upper gastrointestinal tract will become questionable. A decrease of antibiotic use on such a scale may result in a large reduction of needless medical activities, costs, and bacterial resistance. The latter is a growing issue in contemporary medicine and has emerged as one of the eminent public health concerns nowadays [10].

The Perioperative Antibiotic Use in the Treatment of Acute Inflammation of the Gallbladder (PEANUTS II) trial is designed to assess whether preoperative antibiotic prophylaxis is indicated to prevent postoperative infectious complications in patients undergoing emergency cholecystectomy for acute calculous cholecystitis. The hypothesis is that the absence of antibiotic prophylaxis does not lead to an increase in infectious complications.

## Methods

### Design

**Fig. 1:**
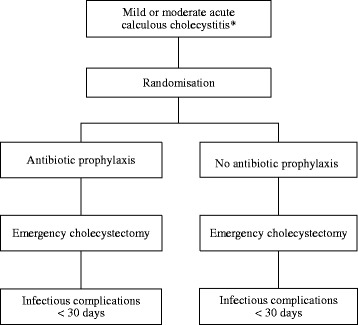
Flowchart of study outline of included patients. * According to the severity assessment criteria of the Tokyo Guidelines [11]

The PEANUTS II trial is a randomized, controlled, multicenter, open-label noninferiority trial. Patients will be randomly allocated to receive either no antibiotics or a single dose of antibiotic prophylaxis before emergency cholecystectomy (Fig. 1).

### Study population

All patients presenting with acute calculous cholecystitis to one of the participating hospitals will be assessed for eligibility. Patients are eligible if diagnosed with mild or moderate acute calculous cholecystitis as defined according the Tokyo Guidelines [11] (Table 1). The inclusion and exclusion criteria are presented in Table 2.

**Table 1:** Diagnostic criteria for acute cholecystitis according to the Tokyo Guidelines [11]

Severity grade	Criteria
Mild (grade I)	Does not meet the criteria of “severe” or “moderate” acute cholecystitis. Can also be defined as acute cholecystitis in a healthy patient with no organ dysfunction and mild inflammatory changes in the gallbladder.
Moderate (grade II)	Associated with any one of the following conditions: 1. Elevated white blood cell count (>18,000/mm^3^) 2. Palpable tender mass in the right upper abdominal quadrant 3. Duration of complaints > 72 h 4. Marked local inflammation (gangrenous cholecystitis, pericholecystic abscess, hepatic abscess, biliary peritonitis, emphysematous cholecystitis)
Severe (grade III)	Associated with dysfunction of any of the following organs/systems: 1. Cardiovascular dysfunction (hypotension requiring treatment with dopamine > 5 μg/kg/minute or any dose of norepinephrine) 2. Neurological dysfunction (decreased level of consciousness) 3. Respiratory dysfunction (PaO_2_/FiO_2_ ratio < 300) 4. Renal dysfunction (oliguria, creatinine > 2.0 mg/dl) 5. Hepatic dysfunction (PT-INR > 1.5) 6. Hematological dysfunction (platelet count < 100,000/mm^3^)

*Abbreviations: PaO*_*2*_ */FiO*_*2*_ Ratio of partial pressure of arterial oxygen to fraction of inspired oxygen, *PT-INR* Prothrombin time-international normalized ratio

**Table 2:** Inclusion and exclusion criteria

Inclusion criteria	Exclusion criteria
● Acute calculous cholecystitis, graded as mild or moderate^a^ according to Tokyo Guidelines [11] *A*. Local signs of inflammation and so forth: 1. Murphy’s sign 2. RUQ mass/pain/tenderness *B*. Systemic signs of inflammation and so forth: 1. Fever 2. Elevated CRP 3. Elevated WBC count * C*. Imaging findings suspect for acute cholecystitis *Definite diagnosis: one positive item in A and one positive item in B* ● Patient will undergo cholecystectomy	● < 18 years of age ● Acalculous cholecystitis ● Acute calculous cholecystitis graded as severe according to Tokyo Guidelines [11] ● Already receiving or needing antibiotics for a concomitant infection ● Proven allergy to cefazolin ● Pregnancy ● Indication for ERCP on admission

*Abbreviations: CRP* C-reactive protein, *ERCP* Endoscopic retrograde pancreaticocholangiography, *RUQ* Right upper quadrant, *WBC* White blood cell

^a^The diagnostic criteria for mild and moderate acute cholecystitis are shown in Table 1

### Primary and secondary endpoints

The primary endpoint is a composite endpoint consisting of all postoperative infectious complications occurring during the first 30 days after surgery. Table 3 provides an overview of the definitions. Secondary endpoints include all the individual components of the primary endpoint and, in addition, all other complications, the total postoperative duration of hospital stay, and the total costs.

**Table 3:** Definitions of various infectious complications

Complication	Definition
Superficial incisional	Localized signs such as redness, pain, heat, or swelling at the site of the incision or by the drainage of pus
Deep incisional	Presence of pus or an abscess, fever with tenderness of the wound, or separation of the edges of the incision exposing the deeper tissues
Organ or space infection	Fever and/or elevated CRP/WBC count and intra-abdominal fluid collection visualized by CT imaging or ultrasound
Pneumonia	Coughing or dyspnea, radiography with infiltrative abnormalities, or elevated infection parameters in combination with positive sputum culture
Urinary tract infection	Dysuria, elevated WBC count, and/or presence of nitrate in urine sediment in combination with a positive urine culture
Bacteremia	Presence of at least one positive hemoccult test result for the same pathogen

*Abbreviations: CRP* C-reactive protein, *CT* Computed tomographic, *WBC* White blood cell

### Randomization

Patients will be randomly assigned to the treatment group (antibiotic prophylaxis) or the nontreatment group (no antibiotic prophylaxis) as shown in the flowchart in Fig. 1. Randomization is performed using an online randomization module (ALEA2.2, https://nl.tenalea.net/amc/ALEA/; Academic Medical Center, Amsterdam, The Netherlands) and stratified according to center. Computer-generated permuted block randomization with varying block sizes is being used with a maximum block size of six patients. The sequence of the different blocks is predetermined by an independent programmer and concealed to all investigators.

### Treatment protocol

#### Preoperative management

To confirm the diagnosis, all patients presenting with suspected acute calculous cholecystitis will undergo standard laboratory workup and ultrasound examination of the abdomen or contrast-enhanced computed tomography if ultrasound results are inconclusive. When patients are eligible for inclusion and informed consent is obtained, randomization will take place. Patients in the treatment group will receive 2000 mg of first-generation cephalosporin administered intravenously 15–30 minutes before surgery. Patients in the nontreatment group will not receive any antibiotic prophylaxis. Cholecystectomy should be performed within 24 h after randomization. Figure 1 demonstrates the study outline of patient inclusion.

#### Surgical management

Cholecystectomy will be performed laparoscopically using the four-trocar technique according to the guidelines of the Dutch Society of Surgery, which includes the critical view of safety techniques [12]. The surgical procedure will be performed by or under the supervision of an experienced laparoscopic surgeon.

#### Postoperative management

Patients will be discharged on the basis of their clinical condition and at the discretion of the treating physician. If patients in either group develop infectious complications, antibiotic therapy will be started. All events will be recorded.

### Data collection and follow-up

**Fig. 2:**
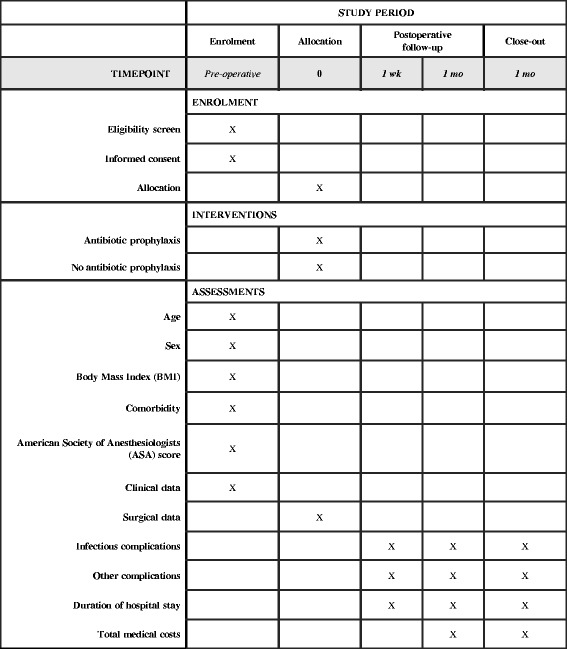
Content for schedule of enrollment, interventions, and assessments according to the Standard Protocol Items: Recommendations for Interventional Trials statement [13]

Each patient will receive an anonymous study number that will be used for the study record forms and the database. On admission, baseline characteristics, including age, sex, body mass index, comorbidity, American Society of Anesthesiologists physical status score, clinical data (i.e., temperature on admission, white blood cell count, C-reactive protein, and duration of symptoms), will be collected and documented by the admitting physician or (local) study coordinator (Fig. 2). Data regarding the surgical procedure, including conversion, bile culture, empyema, bile spill, and the severity of cholecystitis, will be documented by the performing surgeon immediately after the procedure. On the day of discharge, a case record form will be completed with information on the occurrence of infectious complications and, if so, the way the infection was objectified and treated. One week after discharge, the patients will be called by phone by the study coordinator, and 1 month after discharge, the patient will be seen in the outpatient clinic by a surgeon who will complete a questionnaire on the patient’s clinical condition and the development of infectious complications. Every 3 months, all entered data will be checked for completion by the study coordinator, and missing data will be collected from the participating centers.

### Safety

A data and safety monitoring board (DSMB) consisting of three independent members has been appointed to assess patient safety. The first meeting will take place after 20 patients are included and subsequently once per every 50 included patients. The DSMB has unblinded access to all data.

### Adverse events

An adverse event is defined as an undesirable experience of a subject during the study, regardless of whether it is considered related to the intervention. Participating physicians will report all adverse events to the study coordinator immediately on occurrence. The study coordinator will list all adverse events and will present these to the DSMB for every 30 randomized patients. All adverse events will be reported to the Dutch Central Committee on Research Involving Human Subjects using the committee’s online module (http://www.toetsingonline.nl).

### Ethics

The PEANUTS II trial is being conducted in accordance with the Declaration of Helsinki and Dutch law regarding research involving human subjects (Wet Medisch Wetenschappelijk Onderzoek met Mensen). The study protocol (number NL53084.100.15, version 1.0) was approved by the Medical Research Ethics Committee (MEC-U) of the St. Antonius Hospital, Nieuwegein, The Netherlands, on November 24, 2015. Secondary approval was obtained from the executive boards of all participating centers. The study protocol was retrospectively registered (after enrollment of the first participant) with the Netherlands Trial Register at www.trialregister.nl (registration number NTR5802) on June 4, 2016. Written informed consent will be obtained from each participant before any trial-related procedures are carried out.

The present study protocol is written according to the Standard Protocol Items: Recommendations for Interventional Trials (SPIRIT) 2013 statement for reporting a clinical trial protocol [13]. The SPIRIT checklist is provided in Additional file 1.

### Statistical considerations

#### Sample size calculation

A recently published randomized controlled trial showed a postoperative infectious complication rate of 17% in patients with mild and moderate acute calculous cholecystitis undergoing laparoscopic cholecystectomy [1]. This percentage has been used as the reference number for the sample size calculations of the PEANUTS II trial. A noninferiority margin of 10% is assumed. This figure is based on recommendations of the U.S. Food and Drug Administration, which recommends 10% for anti-infective trials. With a 1-sided risk of 2.5% and a power of 80%, a total of 454 subjects will have to be included in the trial.

#### Descriptive statistics

Dichotomous data and counts will be presented as frequencies. Continuous data will be presented as means with standard deviations or, in cases of skewed distribution, as medians with interquartile ranges.

#### Analyses

After the last patient has completed follow-up, raw data will be presented to an adjudication committee to determine whether the endpoints meet the protocol-specified criteria. Each member of the adjudication committee is blinded to the treatment allocation and will assess the potential endpoints individually. Disagreement will be resolved in a plenary consensus meeting. After consensus is reached on each individual endpoint for each patient, final analysis will be performed by an unblinded independent statistician. This analysis will be performed according to the intention-to-treat principle, which means that all randomized patients will be included in their initially assigned study arm, regardless of adherence to the protocol.

For nominal data, the chi-square test or Fisher’s exact test will be used; for continuous data, the Mann-Whitney *U* test will be used. Noninferiority will be demonstrated if the upper limit of the two-sided 95% CI of the difference of the proportion of the primary endpoint between the two groups is lower than the noninferiority margin. The effect will be measured by the absolute risk difference, and the precision will be quantified by means of the 95% CI. The formal statistical hypothesis regarding noninferiority will be tested by the Westlake-Schuirmann test, with the noninferiority margin set at 10% and a one-tailed *p* value < 0.025 considered statistically significant. For all other tests, a two-tailed *p* value < 0.050 is considered statistically significant. In general, for the primary endpoint, we do not expect data to be missing; if so, missing data will not be imputed.

#### Premature termination of the study

The DSMB will perform a formal interim analysis for superiority with respect to the primary endpoint when 50% (*n* = 227) of the total number of patients have been randomized and have completed the 1-month follow-up. The Peto approach will be followed, which includes that the study will be stopped only for benefit or harm in case of a *p* value < 0.001 [14]. Because this is the first randomized trial on this subject and because future treatment policy will be based on it, this trial will not be stopped for futility.

#### Feasibility

Recruitment commenced in March 2016 and is anticipated to run until March 2019. Currently, patients are being recruited at seven major teaching hospitals in The Netherlands. Every 6 months, the inclusion rate will be assessed. If accrual is too slow, additional centers will be invited to participate.

## Discussion

Whether antibiotic prophylaxis has any additional value in preventing infectious complications after emergency cholecystectomy is a much-debated subject in the surgical community. Evidence-based guidelines are lacking, and, as a result, the use of antibiotic prophylaxis varies greatly among surgeons and hospitals. Recently, high-level evidence became available demonstrating that *postoperative* antibiotic prophylaxis (in addition to a single preoperative dose) does not reduce the risk of infectious complications [1]. *Preoperative* antibiotic prophylaxis in relation to the risk of infectious complications, however, has never been studied. We therefore designed the PEANUTS II trial with the aim of determining the utility of preoperative antibiotic prophylaxis in patients undergoing emergency cholecystectomy for acute calculous cholecystitis.

The PEANUTS II trial has a noninferiority design. The hypothesis is that the absence of antibiotic prophylaxis will not lead to an increase of postoperative infectious complications. In the nontreatment group, either an increase of the infectious complication rate or no effect will be seen. Because it is very improbable that the absence of antibiotic prophylaxis will lead to a decrease of infectious complications, and thus deviation is possible in only one direction, a noninferiority design is best suited to answering this primary question.

The best design for a therapeutic trial is a placebo-controlled, double-blind trial, but because treatment of acute cholecystitis is often performed in an acute setting outside regular working hours, such a design is difficult to organize. Therefore, an open comparative design was chosen. We believe that blinding and placebo are not of absolute importance, because the primary outcome of the study is an objective criterion with a clearly defined (internationally accepted) definition. In addition, all potential endpoints will be assessed individually by the members of the adjudication committee, who are blinded to the treatment allocation. Only after reaching consensus on each individual endpoint for each patient will final analysis be performed by an unblinded independent statistician.

Patients with grade III (severe) acute cholecystitis are septic and require antibiotic treatment in addition to appropriate organ support [11, 15]. This is the rationale for exclusively including patients with grade I (mild) and grade II (moderate) acute cholecystitis.

If this study demonstrates that omitting antibiotic prophylaxis does not increase the postoperative infection rate in patients with acute cholecystitis, the role of antibiotic prophylaxis in surgery of the entire upper gastrointestinal tract will become questionable. A decrease in the use of antibiotics may result in a large reduction of bacterial resistance, the latter being an increasingly serious threat to global public health [10].

### Trial status

Recruitment commenced in March 2016 and is anticipated to run until March 2019. As of August 20, 2017, 158 patients had been randomized, and 7 hospitals were participating in the trial. The study results will be communicated via publication.

## Additional file

Additional file 1: SPIRIT (Standard Protocol Items: Recommendations for Interventional Trials) checklist. (DOCX 51 kb)

## Abbreviations

CRP: C-reactive protein
CT: Computed tomography
DSMB: Data and safety monitoring board
ERCP: Endoscopic retrograde pancreaticocholangiography
PaO_2_/FiO_2_: Ratio of partial pressure of arterial oxygen to fraction of inspired oxygen
PEANUTS II: Perioperative Antibiotic Use in the Treatment of Acute Inflammation of the Gallbladder
PT-INR: Prothrombin time-international normalized ratio
RUQ: Right upper quadrant
SPIRIT: Standard Protocol Items: Recommendations for Interventional Trials
WBC: White blood cell
